# Non-destructive morphological observations of the fleshy brittle star, *Asteronyx loveni* using micro-computed tomography (Echinodermata, Ophiuroidea, Euryalida)

**DOI:** 10.3897/zookeys.663.11413

**Published:** 2017-03-27

**Authors:** Masanori Okanishi, Toshihiko Fujita, Yu Maekawa, Takenori Sasaki

**Affiliations:** 1 Faculty of Science, Ibaraki University, 2-1-1 Bunkyo, Mito, Ibaraki, 310-8512 Japan; 2 National Museum of Nature and Science, 4-1-1 Amakubo, Tsukuba, Ibaraki, 305-0005 Japan; 3 University Museum, The University of Tokyo, 7-3-1 Hongo, Bunkyo, Tokyo, 113-0033 Japan

**Keywords:** Anatomy, Asteronychidae, computed tomography, Euryalida, soft tissue, taxonomy

## Abstract

The first morphological observation of a euryalid brittle star, *Asteronyx loveni*, using non-destructive X-ray micro-computed tomography (µCT) was performed. The body of euryalids is covered by thick skin, and it is very difficult to observe the ossicles without dissolving the skin. Computed tomography with micrometer resolution (approximately 4.5–15.4 µm) was used to construct 3D images of skeletal ossicles and soft tissues in the ophiuroid’s body. Shape and positional arrangement of taxonomically important ossicles were clearly observed without any damage to the body. Detailed pathways inside the vertebral ossicles, lateral arm plates, and arm spines for passage of nerves and water vascular structures were observed. Inter-vertebral muscles were also observed. Forms and 3D arrangements of many important taxonomical characters of the euryalids were scrutinized by µCT in high enough resolution for taxonomic description of ophiuroids.

## Introduction

The class Ophiuroidea (phylum Echinodermata) is globally distributed, ranging from the equator to polar regions, and from the intertidal zone to the greatest depths (Stöhr et al. 2012; O’Hara et al. 2014). Ophiuroidea is the most diverse class in Echinodermata, comprising approximately 2100 species (Stöhr et al. 2012). Among the ophiuroids, the order Euryalida, one of two orders of superorder Euryaophiurida, comprises 186 valid species (Okanishi and Fujita 2013; O’Hara et al. 2017), which accounts for about 10% of the species of Ophiuroidea.

The ophiuroid skeleton is composed of numerous small ossicles whose shapes and sizes have been intensively used for taxonomy of the class Ophiuroidea (e.g., Fell 1960; Smith et al. 1995; Stöhr et al. 2012). Shapes and arrangements of their superficial ossicles, such as oral plates, adoral shields, and arm plates can be relatively easily observed in the order Ophiurida and superorder Ophintegrida which are the ophiuroids other than euryalids, through their thin integuments, but the order Euryalida has a thick skin on their body surface which conceals the ossicles and impairs their external observation (Baker 1980). In Euryalida, presence/absence or density of superficial external ossicles, and length of very conspicuous radial shields have been used as taxonomic characters (e.g., Lyman 1882; Döderlein 1911; Matsumoto 1917), but they cannot usually be observed without removal of their skin. These characters can vary with growth, and different developmental stages of the same species have been mistakenly described as a different species (e.g., *Astrothorax waitei*, see Baker 1980; *Asteronyx loveni*, see Stöhr 2005). Recently, many taxonomists have intentionally removed the skin and observed the various ossicles directly, using the shape and size of each external ossicle, and their arrangements, presence/absence of adoral shields, and layered structure of radial shields as taxonomic characters in Euryalida (e.g., Stöhr 2005; Okanishi and Fujita 2011).

To remove the skin in Euryalida, a solution of sodium hypochlorite has been used which dissolves the epidermis (e.g., Mortensen 1933; Stöhr 2011). As a result, some morphological characters are lost. For example, although intact parts were left after the dissection as ophiuroids are pentaradial, part of the external ossicles and skin of the paratypes of *Asterostegus sabineae* and *Squamophis lifouensis* have been lost when treated with bleach to reveal the deeper embedded ossicles (Stöhr 2011; Okanishi and Fujita 2014).

The ossicles which are deep inside the body have been used for higher-level taxonomic characters of Ophiuroidea (e.g., Matsumoto 1917; Smith et al. 1995). Matsumoto (1917) discussed that shapes and numbers of peristomial plates were diagnostic characters to distinguish his four orders of Ophiuroidea, Chilophiurida, Gnathophiurida, Laemophiurida and Phrynophiurida. The peristomial plates have hardly ever been observed or described by subsequent workers, because destructive dissection of the disc is required for their observation. For example, the disc of the holotype of *Astrophyton annulatum* Matsumoto, 1912 has been split into two halves (see Fujita 2006: Fig. 3D; the picture available also on http://umdb.um.u-tokyo.ac.jp/DDoubutu/invertebrate/ophiuroidea/type.html). Such a destructive method increases the risk of loss of characters, for example jaws, and many taxonomists refrain from applying destructive methods to specimens. As a consequence not only the peristomial plates, but also oral and dental plates as well as genital plates, and other internal disc ossicles have been observed and described in the type specimens of only a limited number of ophiuroid species.

Micro-computed tomography (µCT) is a non-destructive imaging technique using X-ray. This method allows rapid creation of three dimensional (3D) morphological and anatomical images at µm scale resolution of biological materials. The output data can then be analyzed with virtual dissection and with rotation optionally, so that 3D arrangements of complex combinations of materials can be recognized (Faulwetter et al. 2013). Micro-CT technology can be used for dried and wet biological specimens (e.g., ethanol preserved and formalin fixed specimens) and is suitable for imaging of hard materials, such as calcareous skeletons (e.g., Ziegler 2012). While this has been a popular analytical method in paleontology (e.g., Hamada et al. 1991; Hendrickx et al. 2006; Tafforeau et al. 2006; Sutton 2008), the application of µCT to morphology and anatomy of extant invertebrate taxa began only recently (Golding and Jones 2006; Greco et al. 2008; Golding et al. 2009; Heim and Nickel 2010; McPeek et al. 2011; Ziegler et al. 2011; Ziegler 2012; Faulwetter et al. 2013; Kohtsuka 2014; Sentoku et al. 2015; Landschoff and Griffiths 2015).

In the Ophiurida and Ophintegrida, µCT observation has been applied to *Ophiocomina nigra* (Ziegler et al. 2011), *Ophiomastix mixta* and *Ophiarachnella gorgonia* (Kohtsuka 2014) and these authors showed horizontal sections and 3D reconstruction images. These images provide evidence that shapes and arrangements of various ossicles can be clearly illustrated non-destructively. Recently, high resolution 3D visualization was performed to observe brooding behavior in three brittle stars, *Amphiura capensis*, *Amphipholis squamata* and *Ophioderma wahlbergii* (Landschoff and Griffiths 2015; Landschoff et al. 2015; Du Plessiss et al. 2015). They successfully observed the positions and postures of brooded juveniles by 3D construction of CT images, but their descriptions were not sufficient for anatomical and/or taxonomical studies. Until now no µCT observations have been performed on species in the order Euryalida.

*Asteronyx loveni* Müller & Troschel, 1842 is a very fleshy brittle star and it is very difficult to study the skeletal ossicles embedded in its thick skin. To study skeletal elements of this species, destructive anatomical dissection and dissolution of skin have been employed (e.g., Mortensen 1912; Matsumoto 1917). In the present study, for the first time, we applied µCT scanning to the Euryalida, using *Asteronyx loveni* to non-destructively observe ossicle morphology at an enough resolution for taxonomic description. We focused our analysis on the shape and arrangements of external ossicles, oral plates and adoral shields because they have scarcely been observed or described in euryalids but are well described for most species of Ophiurida and Ophintegrida as useful taxonomic characters especially from external views. We observed a single vertebral ossicle and illustrated pathways of radial nerve canals and radial water canals in the ossicle, which have never previously been described. Additionally, we tried to observe soft tissues such as muscles, which have not previously been observed by µCT scanning.

## Materials and methods

### Sample preparations

Applying µCT to an entire specimen, an arm fragment from a second specimen, and an isolated vertebra of *Asteronyx loveni*.

Two specimens of *Asteronyx loveni* deposited in the National Museum of Nature and Science, Japan (NSMT), were selected for examination (Table 1). The entire body of a specimen (NSMT E-6986, disc diameter 5.8 mm) and a basal part of an arm of another specimen (NSMT E-5638, disc diameter 10.7 mm) were air-dried for µCT study. A vertebral ossicle was isolated from the latter specimen by immersion in domestic bleach (approximately 5% sodium hypochlorite solution), washed in deionized water, and dried in air for digital microscope observation and µCT observation. We examined shapes of a variety of skeletal ossicles and their positional arrangements, internal structures of a vertebra and soft tissues.

**Table 1.** Sampling information of the two examined specimens of *Asteronyx loveni*.

**Catalog Number**	**Locality**	**Water Depth (m)**	**Date**
NSMT E-6986	East China Sea, southwestern Japan, 26°56.30'N, 127°37.00'E	648	June 1, 2011
NSMT E-5638	Off Miyako, northeastern Japan, 39°20.19'N, 142°51.39'E; -39°19.22'N, 142°49.17'E	1709-1737	November 6, 2007

Morphological terminology follows Stöhr et al. (2012). Some additional terms for euryalid ophiuroids follow Byrne (1994), Stewart (2000), and Okanishi and Fujita (2014). Especially, we here provide explanation about superficial ossicles of euryalid ophiuroids. Recently, both “epidermal ossicles” and “external ossicles” have been used for those ossicles in descriptions of euryalid ophiuroids (e.g., Okanishi and Fujita 2014; Okanishi 2017). However, “epidermal ossicles” may not be suitable because epidermis is frequently lost in echinoderms. Therefore, we use “external ossicles” for the superficial ossicles in this study.

### µCT observation and 3D reconstructions

A ScanXmate B100TSS110 µCT (Comscantecno Co., Ltd.) was used at the University Museum, The University of Tokyo, Japan. Parameters of scanning are shown in Table 2.

**Table 2.** Scanning parameters of µCT for the observations of *Asteronyx loveni*.

Observed specimen	Source voltage (kV)	Source current (µA)	Exposure time for 1 frame (sec)	Total number of frames	Total time for scanning (min)	Detector size (pixel)	Resolution (µm)
Entire body	80	155	1.0	1,500	25	1,024 × 1,012	15.440
Basal part of an arm	100	100	1.2	1,200	16	1,024 × 1,012	13.759
Isolated vertebral arm ossicle	75	43	0.4	1,200	50	1,024 × 1,012	4.459

3D reconstruction employed Molcer version 1.32 (http://www.white-rabbit.jp/molcer.html) using image stacks of virtual sections. The single section images were selected by using imageJ software 1.48 ver. (Figs 3B–D; 5B–G; 6D–O). Two kinds of 3D reconstructive images were created by Molcer 1.32: volume rendering and surface rendering. The rendering technique is the computer algorithm used to transform serially acquired CT image data into 3D images. The volume and surface rendering techniques project the 3D data into the 2D viewing plane from the desired point of view (Rodt et al. 2006). Volume rendering examines the intensity of the objects and the rendering images show all projected materials including internal structures. The surface rendering treats the isosurface from the voxel data. This technique created 3D images composed of polygons, and use sharply shading to show the location of a light source. Thus the surface rendered images only show surficial information for each object, but they enable us to recognize the forms of the materials more clearly. Both surface and volume rendering images were created from the same set of scans. All section images were non-destructively obtained by using “virtual dissection” mode of Molcer version 1.32. Management and storage of CT data was implemented in Morphobank (project 2440, http://www.morphobank.org/; O’Leary and Kaufman 2012). This “project” is to store the images of this paper and not public one.

### Microscopic observations

The specimens were also examined by digital microscopes after µCT observations. The entire specimen and a part of arm were observed with a Keyence VHX 1000. The separated vertebral ossicles were observed and photographed with Keyence VHX D510 using a SEM mode.

### Embedding 3D images into PDFs

Three supplementary PDFs with embedded surface were prepared rendering images of the entire specimen of NSMT E-6986 (Suppl. material 1), a basal portion of an arm (Suppl. material 2) and a vertebra (Suppl. material 3). Molcer version 1.32 and Geomagic Sculpt version V2016.0.38 (3D Systems, Inc.: http://www.geomagic.com/en/legal/patents-sensable) were used in preparing the 3D data in STL and U3D format, respectively. “Interactive object tool” of Acrobat XI Pro (Adobe System Inc.) was used to embed the images into PDFs.

## Results

**Figure 1.**
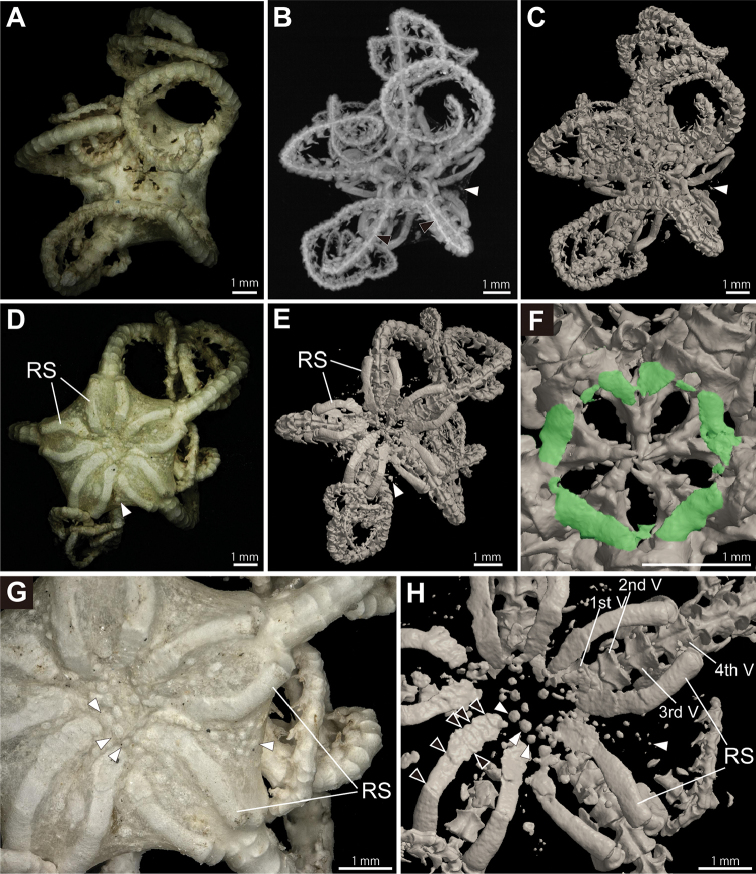
Microscopic (**A, D, G**), µCT volume rendered (**B**) and µCT surface rendered (**C, E, F, H**) images of the entire body of *Asteronyx loveni* (NSMT E-6986). **A–C** whole animal, oral view. Black arrow heads indicate longitudinal dorsal midlines of vertebrae and white arrows indicate external ossicles embedded in skin **D, E** whole animal, aboral view **F** mouth frame, virtually dissecting aboral view, peristomial plates are colored green **G, H** disc, aboral view. White arrow heads indicate external ossicles and black arrows indicate positions where components of plate shaped ossicles of the radial shields are layered. Oral side of bodies were virtually dissected (H). Abbreviations: RS, radial shields; V, vertebra.

**Figure 2.**
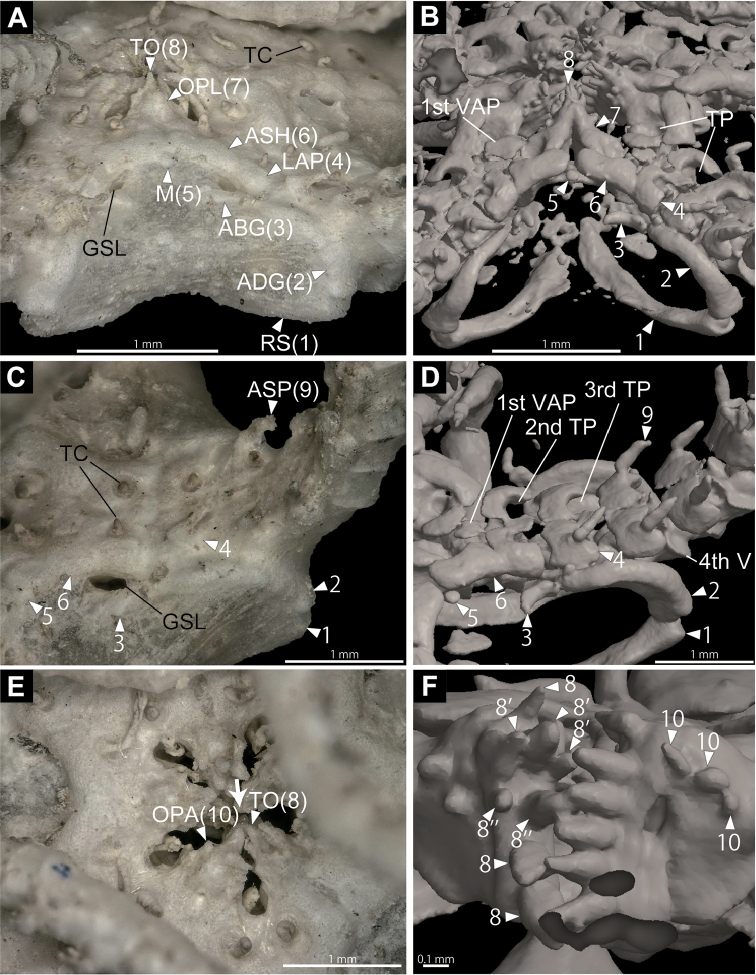
Microscopic (**A, C, E**) and µCT surface rendered (**B, D, F**) images of the entire body of *Asteronyx loveni* (NSMT E-6986). **A, B** a part of disc, oral lateral view **C, D** basal part of an arm, oral lateral view **E** oral disc, oral view. An arrow in **E** indicates the orientation of observation of **F**; **F** two jaws, lateral view. The other jaws are virtually dissected. Parallel teeth are indicated by numeral with apostrophe or double apostrophe. Abbreviations: ABG, abradial plate; ADG, adradial plate; ASH, adoral shield; ASP, arm spine; GSL, genital slit; LAP, lateral arm plate; M, madreporite; OPA, oral papilla; OPL, oral plate; RS, radial shield; TC, tentacle; TO, tooth; TP, tentacle pore; V, vertebra; VAP, ventral arm plate.

**Figure 3.**
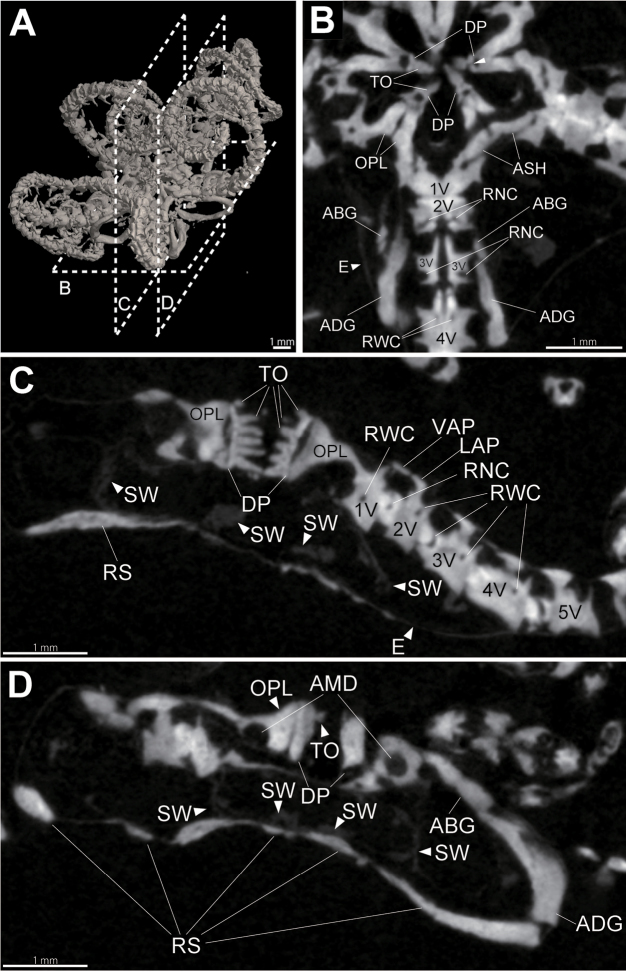
Micro CT surface rendered (**A**) and 2D section **(B–D)** images of the entire body of *Asteronyx loveni* (NSMT E-6986). **A** oral view, section planes of figures **B–D** are shown by dotted tetragons **B** disc and basal part of an arm, horizontal section, a white arrow head indicates vertical crack of a dental plate, a black arrow head indicates the indistinct border of a 1st vertebrae and an oral plate **C** disc and basal part of an arm, vertical section of central disc **D** disc and basal part of an arm, vertical section of abradial disc. Abbreviations: AMD, adradial muscular depression; ABG, abradial plate; ADG, adradial plate; ASH, adoral shield; DP, dental plate; ES, external skin; LAP, lateral arm plate; OPL, oral plate; RNC, radial nerve canal; RS, radial shield; RWC, radial water canal; S, stomach; TO, tooth; V, vertebra; VAP, ventral arm plate.

*Entire specimen (NSMT E-6986)*. The overall morphology of almost all ossicles was clearly visible in volume rendered images and surface rendered images (e.g., Figs 1A–E, G, H; 2; 3A–C; Suppl. material 1). Details of each ossicle were more clearly visible on surface rendered images than volume rendered images (Fig. 1B, C). In the aboral view of the surface rendered images, radial shields are clearly observed, not reaching to the disc center (Fig. 1E). Radial shields are clearly multilayered (Fig. 1H). The outer edges of radial shields are located on the abradial side of the 4th vertebra (Fig. 1D, E, G, H). Peristomial plates are observed on the aboral side of each oral frame, situated on the aboral side of the 1st vertebra and oral plates, and are oblong, twice as wide as long (Fig. 1F). Smaller additional peristomial plates are present between the larger peristomial plates, variable in shape and one-third to one-fourth of the peristomial plates in length (Fig. 1F). External ossicles in the skin on the aboral disc are circular or oblong and spheroid-shaped. The diameter of external ossicles are larger in the center of disc and smaller in the peripheral disc, except some large ones in the interradial area (Fig. 1E, H), which are separated from each other.

In the surface rendered image of the oral side of the disc, the outer edges of the radial shields are articulated with the outer edges of the adradial genital plates on the abradial side of the 4th vertebra (Fig. 2B, D). Adradial and abradial genital plates are bar like and latter is much smaller. Adradial and abradial genital plates are articulated on the abradial side of the 3rd vertebra (Fig. 2D). Adoral shields are rectangulare parallelepiped (Fig. 2B), and connect to the first lateral arm plates (Fig. 2B, D). Oral plates are triangular prism, slightly pointed to proximo-oral side, and in contact with each other on the midline of each jaw (Fig. 2B). One small circular madreporite is located on the distal side of the adoral shields (Fig. 2B, D). Spearhead-shaped teeth are situated on the top of the jaw (Fig. 2B, F). Five to six teeth form a vertical row on dental plate, and another parallel row of two or three teeth is also formed in three of five jaws (Fig. 2F). The length of teeth decreases from aboral toward oral side (Fig. 2F). Two or three granule-like oral papillae present on the lateral side of oral plates, and they are not acute, granule-like (Fig. 2F).

Section images are obtained non-destructively (e.g., Fig. 3A, B) and show the positional arrangement of the ossicles, the internal structure of vertebrae, dental plates, and oral plates (Fig. 3B–D). Oral plates are in contact with the first vertebra (Fig. 3B) and the adoral shields are located on the oral side of the oral plates (Fig. 3B). Oblong dental plates are observed on top of the oral plates (Fig. 3B, C). All dental plates are partly vertically fragmented and a vertical crack is observed in one dental plate (Fig. 3B). The adradial genital plates are in contact with the abradial genital plates on the abradial side of the third vertebra (Fig. 3B). An adradial muscular depression is observed on the oral plates (Fig. 3D). External skin and stomach are observed (Fig. 3B–D).

From the surface rendered images, soft tissues such as tentacles are not observed in any µCT images (Fig. 2A–D) and tentacle pore depressions are observed to be formed by lateral arm plate and ventral arm plate (Fig. 2B, D). First to third tentacle pores have no arm spine and fourth or more distal tentacle pores have one, ovoid arm spine (Fig. 2D). The arm spines are approximately half the length of the corresponding arm segment. First ventral arm plate is conspicuous on the third arm segment, ellipse and flat (Fig. 2B, D). From fourth arm segment, rudimentary ventral arm plates are observed and they decreased in size distally.

Stereom structure of the ossicles are not observed but difference in density of ossicles, which depends on the volume of stereom interstices is recognized by volume rendered images (Fig. 1B). For example, the longitudinal median line of vertebrae is more whitish than the other parts of the vertebrae (Fig. 1B). This indicates that stereom with reduced pores and interstices are smaller, and higher density in the median part of vertebrae (Figs 1B; 6D–O). In 2D section images, internal canals of vertebrae are observed (Fig. 3B, C).

**Figure 4.**
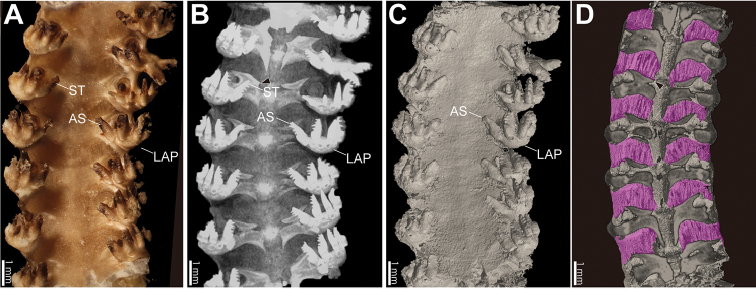
Microscopic (**A**), µCT volume rendered (**B**) and surface rendered (**C, D**) images of basal part of arm (7–13th arm segments) of *Asteronyx loveni* (NSMT E-5638). Oral view. Upper and lower sides indicate distal and basal orientation, respectively. Oral side is virtually dissected in **D** Inter-vertebral muscles are colored purple in **D** Black arrow heads in **B** and **D** indicate identical vertebra, and ST and AS labels also indicates the identical characters in **A, B** and **C**. Abbreviations: ASP, arm spine; LAP, lateral arm plate; ST, secondary teeth.

**Figure 5.**
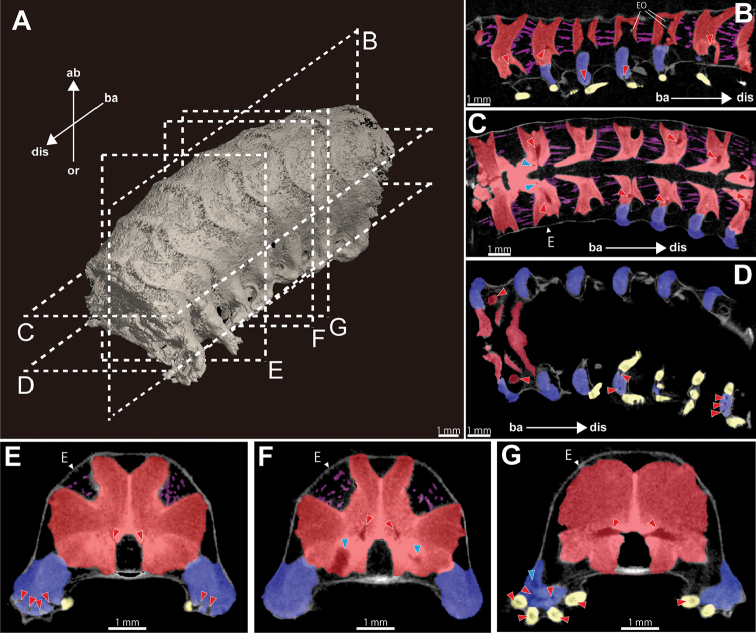
Micro CT surface rendered (**A**) and 2D section (**B–G**) images of the basal part of arm (7–13th arm segments) of *Asteronyx loveni* (NSMT E-5638). **A** basal lateral view. Section planes of figures **B–G** are shown by dotted tetragons **B** vertical longitudinal section, adradial side of the arm **C, D** horizontal longitudinal section, aboral view **C** is set on more aboral side than **D** horizontal longitudinal section, oral view **E–G** transverse vertical sections from basal (**E**) to distal (**G**) arm, basal view. Vertebrae, lateral arm plates, arm spines and muscles are colored red, blue, yellow and purple, respectively. Red and blue arrow heads (**B–G**) indicate radial nerve canals and radial water canals, respectively. Arrows indicate the orientations (ab, aboral; ba, basal; dis, distal; or, oral). Abbreviations: E, epidermis; EO, external ossicle.

*Arm specimen (NSMT E-5638)*. Position of articulation for arm spines of lateral arm plates and detailed shapes of arm spines are observed on volume rendered images without any virtual dissections (Fig. 4A, B), but not on surface rendered images. In the latter case, dried and shrunken thick skin on the arm is detected by X-ray (see also Figs 4D; 5A; Suppl. material 2). The thick skin conceals surface features of the ossicles. Lateral arm plates, arm spines, and vertebrae are observed by 2D section images (Fig. 5B–G). Lateral arm plates are located on the oral lateral side of vertebrae, bar-like, but slightly curved to around the vertebra, approximately twice long wide (Fig. 5B–G). Arm spines are hook-shaped with several (two to five) secondary teeth (Fig, 4B). External ossicles are observed in the skin on the aboral side, small, granule-shaped (Fig. 5B). Inter-vertebral muscles are observed by virtual dissection of surface rendered images (Figs 4D; 5B, C, E, F).

Two pairs of canals are observed inside vertebrae: radial nerve canals and radial water canals (Figs 3B, C; 5B–G). In lateral arm plates, up to five nerve canals are observed on the oral side of each lateral arm plate (Fig. 5D, E, G). The number of arm spines corresponded to the number of nerve canals (Fig. 5B, D, E, G).

**Figure 6.**
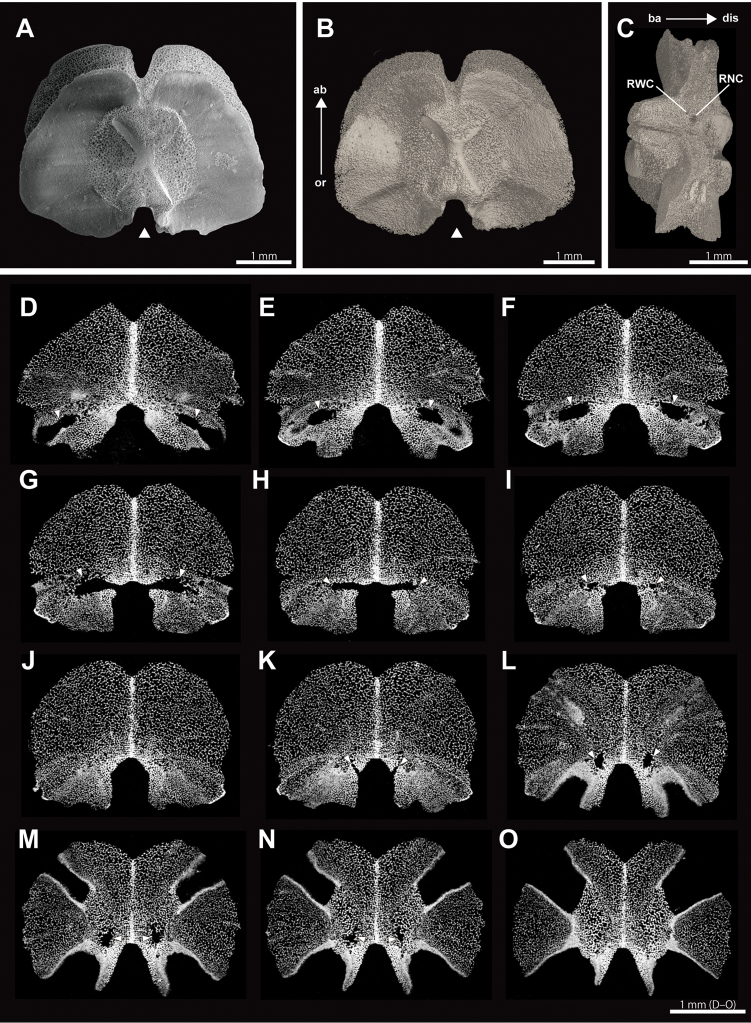
SEM (**A**), µCT surface rendering (**B, C**) and 2D section (**D–O**) images of the isolated vertebral ossicles of *Asteronyx loveni* (NSMT E-5638). **A, B** basal view, arrow heads indicate oral groove **C** oral view **D–O** transverse vertical sections from basal (**D**) to distal (**O**) arm, basal view. Arrow heads indicate radial water canals (**D–I**) and radial nerve canals (**K–O**). Arrows indicate the orientations (ab, aboral; ba, basal; dis, distal; or, oral). Abbreviations: RNC, pore of radial nerve canal; RWC, pore of radial water canal.

*Isolated vertebra (NSMT E5638)*. Moderate resolution (4.5 µm) images are obtained for the isolated vertebra (Fig. 6B–O, Suppl. material 3). Stereom structure is clearly observed in surface rendered images (Fig. 6B). The resolution of the surface rendered images is equivalent to that of SEM images (Fig. 6A). Detailed morphology of radial nerve canals and radial water canals are observed (Fig. 6D–O). A pair of radial water canals opened into the basal part of the oral groove of the vertebra (Fig. 6C; Suppl. material 3), and a radial water canal connected to a depression for tube feet opens on the oral lateral side of the vertebra (Fig. 6D–I). A pair of radial nerve canals opens in a distal position to the oral groove of the vertebra (Fig. 6C). The radial nerve canals extend to the distal side and dead-ended inside the vertebra (Fig. 6K–O).

## Discussion

In the present study, the shapes, numbers, and arrangement of various ossicles of *Asteronyx loveni* were successfully observed by µCT (Figs 1H, 2D, 3, 4B and 5; Suppl. materials 1, 2). The shape, size and arrangement of external ossicles were recently employed as species-level diagnostic characters for euryalids (e.g., Okanishi and Fujita 2009; Okanishi and Fujita 2011), however to examine these characters, once ossicles were extracted by bleaching, their positional relationships to each other could not be obtained. These characteristics of external ossicles were simultaneously observed by µCT without the need to extract them destructively (Fig. 1E, H). Their shape was circular, the size differed depending on the position in the disc, and they were slightly separated from one another (Fig. 1G, H).

Presence of adoral shields is an important diagnostic character of the genus *Asteronyx* (Stöhr 2005). Adoral shields of *Asteronyx* were observed by dissolving the surrounding skin in previous studies (e.g., Mortensen 1912; Stöhr 2005), but they were clearly observed non-destructively in this study (Fig. 2B, D; Suppl. material 1). Two types of ossicle components of radial shields have been known for Euryalida. Astrocharidae has monolayer radial shields and the other families have multilayer radial shields (Okanishi et al. 2011). These shield characters had not previously been recognized without removing the skin with external ossicles of the disc (e.g., Okanishi and Fujita 2009; Stöhr 2011). In this study, µCT observation clearly showed that *Asteronyx loveni* has multilayer radial shields and one radial shield is composed of at least eight plates (Fig. 1H).

Moreover, shapes and 3D positional relationships of radial shields, adradial and abradial genital plates, and the shapes and number of peristomial plates were also successfully observed (Figs 1F; 2A, B). These characters have been considered important for the identification of (sub)orders of Ophiuroidea (e.g., Matsumoto 1917; Smith et al. 1995), but they have been examined for only a very small number of species, and only by destructive dissection. Additional small plates between the peristomial plates were here observed for the first time in Ophiuroidea (Fig. 1F). The above taxonomically important characters of the ossicles have not been confirmed for many type specimens of Ophiuroidea but this study showed they can be easily observed by µCT scanning. This study has shown that µCT is a powerful tool for species- to order-level taxonomy in Ophiuroidea as Ziegler (2012) suggested. Considering that specimens remain non-dissected following µCT observations (Figs 1A, D, G; 2A, C, E; 4A; 6A), this tool is ideal for observing type specimens.

Recently, the micromorphology of the ossicle surface (e.g., articulation forms of lateral arm plates) have become heavily used as taxonomic characters of ophiuroids (e.g., Stöhr et al. 2008; Stöhr and Muths 2010; Okanishi and Fujita 2009, 2011, 2013; Martynov 2010; Thuy and Stöhr 2011; Gondim et al. 2015; Thuy and Stöhr 2016). On the other hand, internal structures of ossicles, such as radial water canals and radial nerve canals in vertebrae, have scarcely been observed and their taxonomic significance has never been discussed. In this study, radial water canals and radial nerve canals in the vertebra, as well as nerve canals in lateral arm plates and arm spines, were observed (Figs 3B, D; 5B–G; 6D–O). The resolution of µCT images in this study (ca. 4.5–15.5 µm) was high enough to observe the density of interstices of stereom structure, and the detailed pathway of radial water canals and radial nerve canals were recognized (Fig. 6D–O). Martynov et al. (2015) observed the canals by serial cross-sections of resin embedded specimens. However, this method is time-consuming and destructive. In the present setting, the total time required for scanning and reconstruction of 3D images ranged from 16 to 50 minutes, much shorter than the time used for cross-sectioning methods.

In this study, inter-vertebral muscles of the dried specimen were observed along with its ossicles (Figs 4D; 5B, C, E, F), which is the first observation of ophiuroid muscles by µCT. Muscles might be increased in density by shrinking when dried, making them detectable by X-ray.

The most novel and remarkable aspects of this study is that complete morphological information of all fundamental ossicles of the order Euryalida was successfully obtained from µCT observations. Micro CT observation has increased the number of available taxonomic characters, which have hardly ever been observed and/or never explored. These taxonomic characters obtained in Euryalida may be compared to those in the order Ophiurida and the superorder Ophintegrida which should accelerate future taxonomic study of the class Ophiuroidea.

## Supplementary materials

Supplementary material 1 Figure S1 Data type: 3D model Explanation note: The interactive 3D model of µ CT surface rendering images of the entire body of *Asteronyx loveni* (NSMT E-6986). This image can be activated by clicking on the image in Adobe Acrobat Reader (version 8 or higher) and can be rotated, moved and magnified. This dataset is made available under the Open Database License (http://opendatacommons.org/licenses/odbl/1.0/). The Open Database License (ODbL) is a license agreement intended to allow users to freely share, modify, and use this Dataset while maintaining this same freedom for others, provided that the original source and author(s) are credited.Masanori Okanishi, Toshihiko Fujita, Yu Maekawa, Takenori Sasaki

Supplementary material 2 Figure S2 Data type: 3D model Explanation note: The interactive 3D model of µ CT surface rendering images of the basal part of an arm of *Asteronyx loveni* (NSMT E-5638). This dataset is made available under the Open Database License (http://opendatacommons.org/licenses/odbl/1.0/). The Open Database License (ODbL) is a license agreement intended to allow users to freely share, modify, and use this Dataset while maintaining this same freedom for others, provided that the original source and author(s) are credited.Masanori Okanishi, Toshihiko Fujita, Yu Maekawa, Takenori Sasaki

Supplementary material 3 Figure S3 Data type: 3D model Explanation note: The interactive 3D model of µ CT surface rendering images of the isolated vertebral ossicles of *Asteronyx loveni* (NSMT E-5638). This dataset is made available under the Open Database License (http://opendatacommons.org/licenses/odbl/1.0/). The Open Database License (ODbL) is a license agreement intended to allow users to freely share, modify, and use this Dataset while maintaining this same freedom for others, provided that the original source and author(s) are credited.Masanori Okanishi, Toshihiko Fujita, Yu Maekawa, Takenori Sasaki

## References

[B1] Baker AN (1980) Euryalinid Ophiuroidea (Echinodermata) from Australia, New Zealand and the south-west Pacific Ocean. New Zealand Journal of Zoology 7: 11–83. https://doi.org/10.1080/03014223.1980.10423763

[B2] Byrne M (1994) Ophiuroidea. In: Harrison FW Chia F-S (Eds) Microscopic Anatomy of Invertebrates, Vol. 14. Echinodermata. Wiley-Liss, New York, 247–343.

[B3] Döderlein L (1911) Über japanische und andere Euryalae. Abhandlungen der Bayerischen Akademie der Wissenschaften 2: 1–123.

[B4] Du Plessis A Griffith CL Landschoff J (2015) 3-dimensional microCT reconstructions of brooding brittle stars. GigaScience Database. https://doi.org/10.5524/100130

[B5] Faulwetter S Vasileiadou A Kouratoras M Dailianis T Arvanitidis C (2013) Micro-computed tomography: Introducing new dimensions to taxonomy. Zookeys 263: 1–45. https://doi.org/10.3897/zookeys.263.4261 10.3897/zookeys.263.4261 PMC3591762 23653515

[B6] Fell HB (1960) Synoptic keys to the genera of Ophiuroidea. Zoology Publications from Victoria University of Wellington 26: 1–44.

[B7] Fujita T (2006) Tokyo-Daigaku Sogo Hakubutsukan Shozou Kumohitode Hyohon ni Tsuite. The University Museum, the University of Tokyo, material reports 62: 135–150. [In Japanese]

[B8] Golding RE Jones AS (2006) Micro-CT as a novel technique for 3D reconstruction of molluscan anatomy. Molluscan Research 27: 123–128.

[B9] Golding RE Ponder WF Byrne M (2009) Three-Dimensional reconstruction of the odontophoral cartilages of Caenogastropoda (Mollusca: Gastropoda) using Micro-CT: Morphology and phylogenetic significance. Journal of Morphology 270: 558–587. https://doi.org/10.1002/jmor.10699 19107810 10.1002/jmor.10699

[B10] Gondim AE Dias TLP Christoffersen ML Stöhr S (2015) Redescription of *Hemieuryale pustulata* von Martens, 1867 (Echinodermata, Ophiuroidea) based on Brazilian specimens, with notes on systematics and habitat association. Zootaxa 3925: 341–360. https://doi.org/10.11646/zootaxa.3925.3.2 25781748 10.11646/zootaxa.3925.3.2

[B11] Greco M Jones A Spooner-Hart R Holford P (2008) X-ray computerized microtomography (MicroCT): A new technique for assessing external and internal morphology of bees. Journal of Apicultural Research 47: 286–291. https://doi.org/10.1080/00218839.2008.11101476

[B12] Hamada T Tateno S Suzuki N (1991) Three dimensional reconstruction of fossils with X-ray CT and computer graphics. Scientific Papers of the College of Arts and Sciences, the University of Tokyo 41: 107–118.

[B13] Heim I Nickel M (2010) Description and molecular phylogeny of *Tethya leysae* sp. nov. (Porifera, Demospongiae, Hadromerida) from the Canadian Northeast Pacific with remarks on the use of microtomography in sponge taxonomy. Zootaxa 2422: 1–21.

[B14] Henderickx H Cnudde V Masschaele B Dierick M Vlassenbroeck J Van-Hoorebeke L (2006) Description of a new fossil *Pseudograpus* (Pseudoscorpiones: Pseudogarypidae) with the use of X-ray micro-CT to penetrate opaque amber. Zootaxa 1305: 41–50.

[B15] Kohtsuka H (2014) Roles of the technical staffs at Misaki Marine Biological Station. Taxa Proceedings of the Japanese Society of Systematic Zoology 36: 24–32. [In Japanese]

[B16] Landschoff J Du Plessis A Griffiths CL (2015) A dataset describing brooding in three species of South African brittle stars, comprising seven high-resolution, micro X-ray computed tomography scans. GigaScience 4: 52. https://doi.org/10.1186/s13742-015-0093-2 10.1186/s13742-015-0093-2 PMC4647331 26579220

[B17] Landschoff J Griffith CL (2015) Three-dimensional visualization of brooding behavior in two distantly related brittle stars from South African waters. African Journal of Marine Science 37(4): 533–541. https://doi.org/10.2989/1814232X.2015.1095801

[B18] Lyman T (1882) Report on the Ophiuroidea dredged by H.M.S. Challenger during the years 1873–1876. Report on the scientific result of the voyage of H.M.S. Challenger during the years 1873–1876 Zoology 5: 1–386.

[B19] Märkel K Röser U (1985) Comparative morphology of echinoderm calcified tissue: Histology and ultrastructure of ophiuroid scales (Echinodermata, Ophiuroida). Zoomorphology 105: 197–207. https://doi.org/10.1007/BF00312157

[B20] Martynov A (2010) Reassessment of the classification of the Ophiuroidea (Echinodermata), based on morphological characters. I. General character evaluation and delineation of the families Ophiomyxidae and Ophiacanthidae. Zootaxa 2697: 1–154.

[B21] Martynov A Ishida Y Irimura S Tajiri R O’Hara T Fujita T (2015) When ontogeny matters: a new Japanese species of brittle star illustrates the importance of considering both adult and juvenile characters in taxonomic practice. PLoS ONE 10(10): e0139463. https://doi.org/10.1371/journal.pone.0139463 10.1371/journal.pone.0139463 PMC4625035 26509273

[B22] Matsumoto H (1917) A monograph of Japanese Ophiuroidea, arranged according to a new classification. Journal of the College of Science, Imperial University of Tokyo 38: 1–408.

[B23] McPeek MA Symes LB Zong DM McPeek CL (2011) Species recognition and patterns of population variation in the reproductive structures of a damselfly genus. Evolution 65: 419–428. https://doi.org/10.1111/j.1558-5646.2010.01138.x 20874736 10.1111/j.1558-5646.2010.01138.x

[B24] Mortensen T (1912) Über *Asteronyx loveni* M. Tr. Zeitschrift für wissenschaftliche Zoologie 101: 264–289.

[B25] Mortensen T (1933) Studies of Indo-Pacific euryalids. Videnskabelige Meddelelser fra Naturhistorisk Forening i København 96: 1–75.

[B26] O’Hara TD Hugall AF Thuy B Moussalli A (2014) Phylogenetic resolution of the class Ophiuroidea unlocks a global microfossil record. Current Biology 24: 1874–1879. https://doi.org/10.1016/j.cub.2014.06.060 25065752 10.1016/j.cub.2014.06.060

[B27] O’Hara TD Hugall AF Thuy B Stöhr S Martynov AV (2017) Restructuring higher taxonomy using broad-scale phylogenomics: The living Ophiuroidea. Molecular Phylogenetics and Evolution 107: 415–430. https://doi.org/10.1016/j.ympev.2016.12.006 27940329 10.1016/j.ympev.2016.12.006

[B28] Okanishi M Fujita T (2009) A new species of *Asteroschema* (Echinodermata: Ophiuroidea: Asteroschematidae) from southwestern Japan. Species Diversity 14: 115–129.

[B29] Okanishi M Fujita T (2011) A taxonomic review of the genus *Astrocharis* Koehler (Echinodermata: Ophiuroidea: Asteroschematidae) with a description of a new species. Zoological Science 28: 148–157. https://doi.org/10.2108/zsj.28.148 21303207 10.2108/zsj.28.148

[B30] Okanishi M O’Hara TD Fujita T (2011) Molecular phylogeny of the order Euryalida (Echinodermata: Ophiuroidea), based on mitochondrial and nuclear genes. Molecular Phylogenetics and Evolution 61: 392–399. https://doi.org/10.1016/j.ympev.2011.07.003 21798356 10.1016/j.ympev.2011.07.003

[B31] Okanishi M Fujita T (2013) Molecular phylogeny based on increased number of species and genes revealed more robust family-level systematics of the order Euryalida (Echinodermata: Ophiuroidea). Molecular Phylogenetics and Evolution 69: 566–580. https://doi.org/10.1016/j.ympev.2013.07.021 23906601 10.1016/j.ympev.2013.07.021

[B32] Okanishi M Fujita T (2014) A taxonomic review of the genus *Asterostegus* (Echinodermata: Ophiuroidea: Euryalidae). European Journal of Taxonomy 76: 1–18.

[B33] Okanishi M (2017) Zootaxa 4227(4): 543–553. https://doi.org/10.11646/zootaxa.4227.4.4 10.11646/zootaxa.4227.4.4 28187565

[B34] O’Leary MA Kaufman SG (2012) MorphoBank 3.0: Web application for morphological phylogenetics and taxonomy. http://www.morphobank.org [Accessed in 2016 June 8]

[B35] Rodt T Bartling SO Zajaczek JE Vafa MA Kapapa T Majdani O Krauss JK Zumkeller M Matthies H Becker H Kaminsky J (2006) Evaluation of surface and volume rendering in 3D-CT of facial fractures. Dentomaxillofacial Radiology 35: 227–231. https://doi.org/10.1259/dmfr/22989395 16798916 10.1259/dmfr/22989395

[B36] Sentoku A Morisaki H Matsumoto S Ohno R Tomiyama T Ezaki Y (2015) Internal skeletal analysis of the clonial azooxanthellate scleractinian *Dendrophyllia cribrosa* using microfocus X-ray CT images: Underlying basis for its rigid and highly adaptive colony structure. Journal of Structural Biology 189: 37–43. https://doi.org/10.1016/j.jsb.2014.11.002 25463019 10.1016/j.jsb.2014.11.002

[B37] Smith AB Paterson GLJ Lafay B (1995) Ophiuroid phylogeny and higher taxonomy: Morphological, molecular and palaeontological perspectives. Zoological Journal of the Linnean Society 114: 213–243. https://doi.org/10.1111/j.1096-3642.1995.tb00117c.x

[B38] Stewart B (2000) Anatomical features of the euryalid snake star *Astrobrachion constrictum* (Ophiuroidea; Asteroschematidae). Invertebrate Biology 119: 222–233. https://doi.org/10.1111/j.1744-7410.2000.tb00009.x

[B39] Stöhr S (2005) Who’s who among baby brittle stars (Echinodermata: Ophiuroidea): postmetamorphic development of some North Atlantic forms. Zoological Journal of the Linnean Society 143: 543–576. https://doi.org/10.1111/j.1096-3642.2005.00155.x

[B40] Stöhr S Conand C Boissin E (2008) Brittle stars (Echinodermata: Ophiuroidea) from La Réunion and the systematic position of *Ophiocanops* Koehler, 1922. Zoological Journal of the Linnean Society 153: 545–560. https://doi.org/10.1111/j.1096-3642.2008.00401.x

[B41] Stöhr S Muths G (2010) Morphological diagnosis of the two genetic lineages of *Acrocnida brachiate* (Echinodermata: Ophiuroidea) with description of a new species. Journal of the Marine Biological Association of the United Kingdom 90(4): 831–843. https://doi.org/10.1017/S0025315409990749

[B42] Stöhr S (2011) New records and new species of Ophiuroidea (Echinodermata) from Lifou, Loyalty Islands, New Caledonia. Zootaxa 3089: 1–50.

[B43] Stöhr S O’Hara TD Thuy B (2012) Global diversity of brittle stars (Echinodermata: Ophiuroidea). PLoS ONE 7(3): e31940. https://doi.org/10.1371/journal.pone.0031940 10.1371/journal.pone.0031940 PMC3292557 22396744

[B44] Sutton MD (2008) Tomographic techniques for the study of exceptionally preserved fossils. Proceedings of the Royal Society B-Biological Science 275: 1587–1593. https://doi.org/10.1098/rspb.2008.0263 10.1098/rspb.2008.0263 PMC2394564 18426749

[B45] Tafforeau P Boistel R Boller E Bravin A Brunet M Chaimanee Y Cloetens P Feist M Hoszowska J Jaeger JJ Kay RF Lazzari V Marivaux L Nel A Nemoz C Thibault X Vignaud P Zambler S (2006) Applications of X-ray synchrotron microtomography for non-destructive 3D studies of paleontological specimens. Applied Physics A 83: 195–202. https://doi.org/10.1007/s00339-006-3507-2

[B46] Thuy B Stöhr S (2011) Lateral arm plate morphology in brittle stars (Echinodermata: Ophiuroidea): new perspectives for ophiuroid micropalaeontology and classification. Zootaxa 3013: 1–47.

[B47] Thuy B Stöhr S (2016) A new morphological phylogeny of the Ophiuroidea (Echinodermata) accords with molecular evidence and renders microfossils accessible for cladistics. PLoS ONE 11(5): e0156140. doi: 10.1371/journal.pone.0156140 10.1371/journal.pone.0156140 PMC4882042 27227685

[B48] Ziegler A Kunth M Mueller S Bock C Pohmann R Schröder L Faber C Giribet G (2011) Application of magnetic resonance imaging in zoology. Zoomorphology 130: 227–254. https://doi.org/10.1007/s00435-011-0138-8

[B49] Ziegler A (2012) Broad application of non-invasive imaging techniques to echinoids and other echinoderm taxa. Zoosymposia 7: 53–70.

