# Supplementary figures and images for: Non-destructive morphological observations of the fleshy brittle star, Asteronyx loveni using micro-computed tomography (Echinodermata, Ophiuroidea, Euryalida)
Source: Zookeys. 2017 Mar 27;(663):1–19. doi: 10.3897/zookeys.663.11413 (PMC5523172; doi:10.3897/zookeys.663.11413)

Supplementary Figure 1

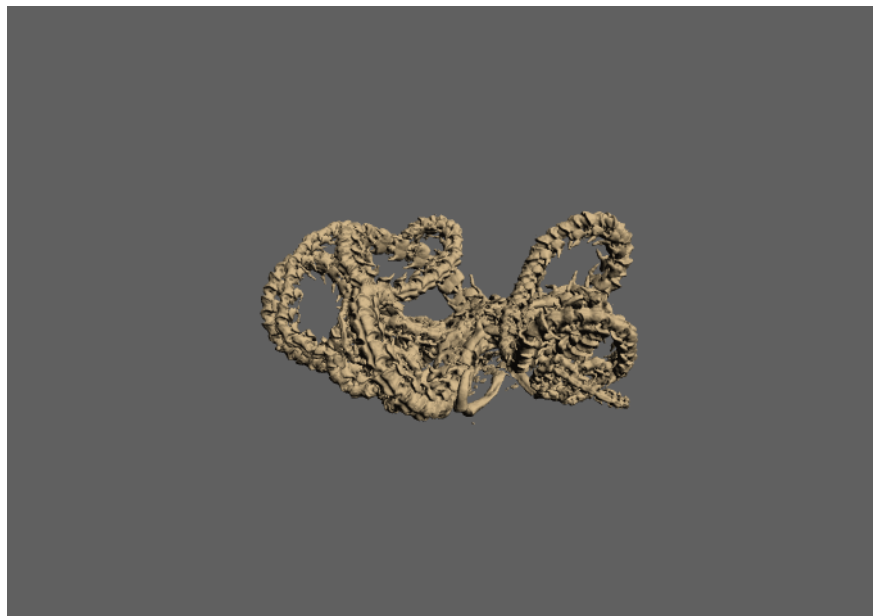

Supplement: Supplementary material 1 — Figure S1 [file zookeys-663-001-s001.pdf]

Supplementary Figure 2

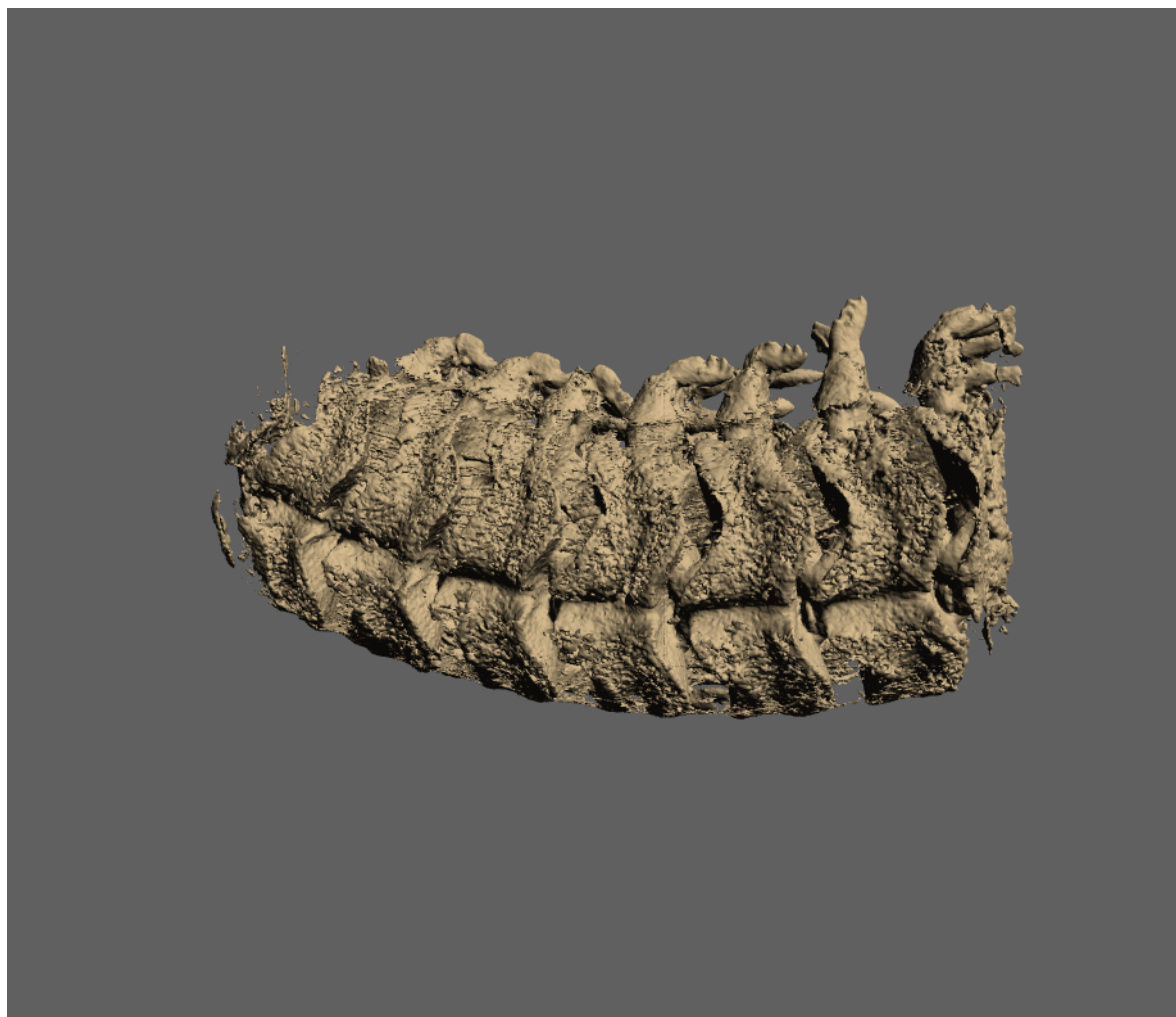

Supplement: Supplementary material 2 — Figure S2 [file zookeys-663-001-s002.pdf]

Supplementary Figure 3

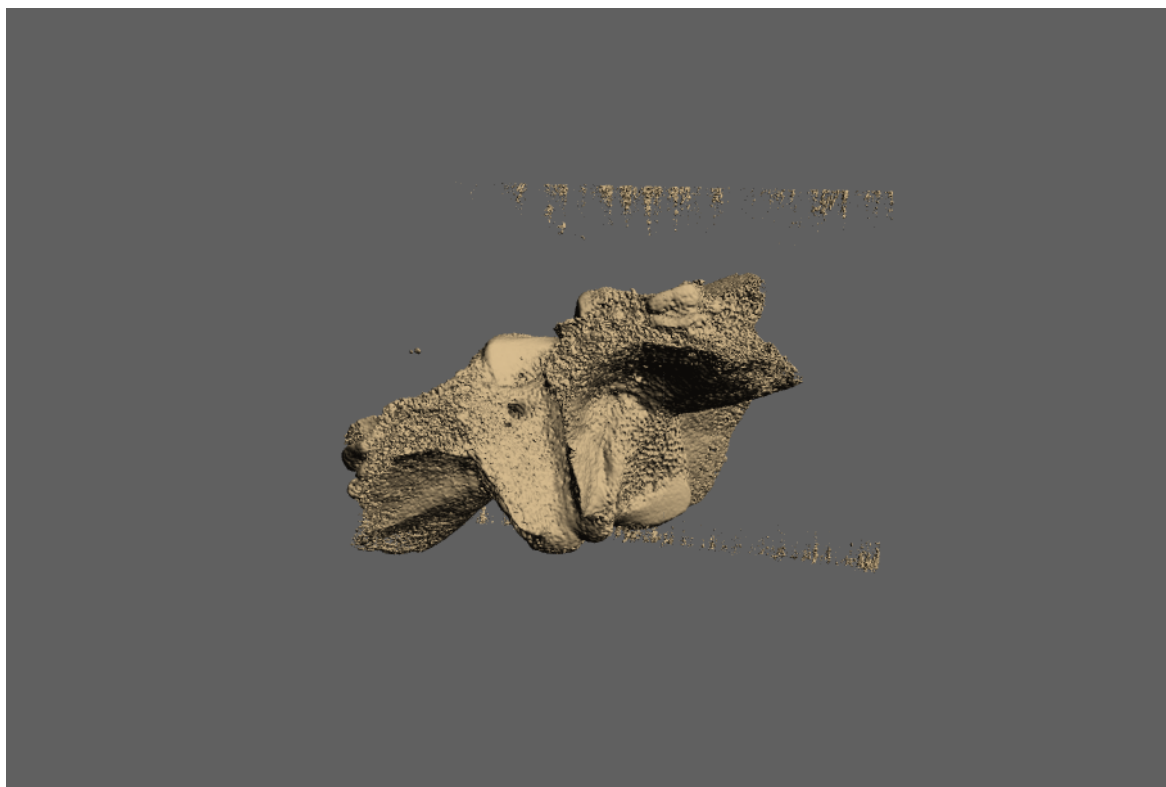

Supplement: Supplementary material 3 — Figure S3 [file zookeys-663-001-s003.pdf]
